# Epidemiology and Evolution of Rotaviruses and Noroviruses from an Archival WHO Global Study in Children (1976–79) with Implications for Vaccine Design

**DOI:** 10.1371/journal.pone.0059394

**Published:** 2013-03-25

**Authors:** Lauren A. Rackoff, Karin Bok, Kim Y. Green, Albert Z. Kapikian

**Affiliations:** Laboratory of Infectious Diseases (LID), National Institute of Allergy and Infectious Diseases (NIAID), National Institutes of Health (NIH), Bethesda, Maryland, United States of America; Tulane University, United States of America

## Abstract

Prompted by the discovery of new gastrointestinal viruses, the NIH, NIAID and WHO investigated the etiology of acute diarrhea that occurred from 1976–1979 in a global cohort of infants and young children. Rotaviruses were found to be major pathogens worldwide, whereas the Norwalk virus could not be detected using a radioimmunoassay. The aim of this study is to re-evaluate the role and diversity of rotaviruses and noroviruses in the original cohort using more sensitive current technologies. Stools collected from Asia, Africa, and South America (n = 485) were evaluated for viral genotypes by RT-PCR and sequencing. Rotaviruses were detected in 28.9% and noroviruses in 9.7% of the specimens, with G1 rotaviruses and GII noroviruses accounting for the majority of each respective virus. Various strains in this study predated the currently assigned dates of discovery for their particular genotype, and in addition, two noroviruses (KL45 and T091) could not be assigned to current genotypes. Phylogenetic analyses demonstrated a relative constancy in circulating rotavirus genotypes over time, with several genotypes from this study becoming established in the current repertoire of viral species. Similarly, GII noroviruses have maintained dominance, with GII.4 noroviruses continuing as a predominant genotype over time. Taken together, the complex molecular epidemiology of rotaviruses and noroviruses circulating in the 1970’s is consistent with current patterns, an important consideration in the design of multivalent vaccines to control these viruses.

## Introduction

Diarrhea has consistently been described as the second leading cause of mortality among children under five years old worldwide [1]. Rotaviruses are the single most important agents of acute diarrhea associated with mortality in this age group, as these viruses were estimated to cause approximately 453,000 deaths a year, mostly in countries of Africa and Asia [2]. Noroviruses are the second leading cause of viral gastroenteritis in children under five years old, and are estimated to cause as many as 200,000 deaths annually among children in this age group in developing countries [3].

Rotaviruses belong to the family *Reoviridae*, and are classified into three groups: A, B, and C, with group A being most commonly associated with gastroenteritis in infants and young children. Group A rotaviruses have a segmented dsRNA genome, which encodes in part for two antigenic outer capsid proteins that are used for viral classification: VP7 (G-type [glycoprotein]) and VP4 (P-type [protease-sensitive protein]) [4]. There are 27 VP7 genotypes and 35 VP4 genotypes that can infect animals and/or humans [5]. The most prevalent G-types that infect humans globally are characteristically G1 through G4, with G9 and G12 emerging in prevalence and G8 and G10 more geographically restricted [4], [6]–[9]. Rotavirus vaccines have significantly reduced the disease burden in Europe, Australia, and the USA, therefore the World Health Organization (WHO) now recommends the inclusion of rotavirus vaccines in childhood vaccine schedules globally [10]. However, rotavirus vaccines are less efficacious in developing countries [11], which demonstrates the need for improved preventive and therapeutic strategies in areas where the disease burden is greatest.

Noroviruses, of the family *Caliciviridae*, have ssRNA positive-sense genomes with three open reading frames. ORF1 encodes a large nonstructural polyprotein that includes the viral RNA-dependent RNA polymerase (RdRp), ORF2 encodes the major capsid protein VP1, and ORF3 encodes the minor capsid protein VP2 [12]. Noroviruses are categorized into Genogroups (G) I through V, with GI, GII, and GIV infecting humans [13]. Currently, there are 8 widely-accepted genotypes in GI and 17 in GII, all of which have been associated with human infection [13]. Genogroup II has the highest circulating frequency, and GII.4 generally predominates as the most commonly-detected genotype in epidemic norovirus gastroenteritis [12], [14]–[16]. Currently, there are no vaccines or antivirals available to prevent or treat norovirus gastroenteritis.

Soon after the discovery of noroviruses in 1972 [17] and rotaviruses in 1973 [18], the NIAID Laboratory of Infectious Diseases (LID) of the National Institutes of Health collaborated with the WHO to determine the role of viruses in childhood diarrhea globally, particularly in developing countries [19]. The LID examined 438 fecal specimens obtained between 1976 and 1979 from infants and young children (including 2 adults) with diarrhea for the presence of rotaviruses and the Norwalk agent, the first detected norovirus genotype, GI.1 [17], [19]. Rotaviruses were detected by an enzyme-linked immunosorbent assay in 27% (n = 118) of the cohort, which was consistent with the WHO recommendation for rotavirus vaccine development [19]–[21]. However, a radioimmunoassay developed for the detection of GI.1 Norwalk virus failed to detect any norovirus strains [19], [22].

The availability of new diagnostic assays now makes it possible to detect a wide range of viral genotypes with a high degree of sensitivity [23]–[29]. The aim of this study was to reevaluate the archival specimens from this global cohort, supplemented by 47 additional archived WHO specimens that were not previously tested, for the presence of rotaviruses and noroviruses. Furthermore, we examined the geographical distribution of previously circulating viral genotypes, while using phylogenetic analyses to assess genomic variation and evolution over a span of more than three decades. Our data confirm the predominance of the rotaviruses that launched concerted vaccine development efforts soon after their discovery, but show also an early underestimation of noroviruses as serious pediatric pathogens. This retrospective approach to rotavirus and norovirus molecular epidemiology and evolution provides insight for the prediction of epidemiologic trends that are relevant to the development of efficacious vaccines for viral gastroenteritis.

## Methods

### Study Cohort and Specimens

Diarrheic stools from 483 infants and young children and 2 adults from the following regions were tested for rotaviruses and noroviruses: Central African Republic, French Guiana, Senegal, Uganda, China, South Korea, Malaysia, Sri Lanka, Tunisia, Democratic Republic of the Congo, and Singapore (Table 1). Collection dates ranged from 1976 to 1979, and ages of individuals, reported for 312 children, ranged from 3 weeks to 14 years old, with the majority between 3 weeks and 3 years of age (n = 284). Hospitalization status was reported for 266 of 483 children; 259 (97.4%) of the 266 children were hospitalized. Stools were transported on dry ice by air to the WHO headquarters in Geneva, Switzerland where they were stored and later sent on dry ice by air to the NIH. Stools were stored upon arrival at approximately −80°C, and diluted into 2% suspension filtrates in PBS for testing.

**Table 1:** Distribution of Viral Genotypes Detected in Each Geographic Region.

Location	Number of Patients Studied	Number of Patients RotavirusPositive (%)	Rotavirus Genotypes Detected and Number (n)	Number of Patients Norovirus Positive (%)	Norovirus Genotypes Detected and Number (n)^b^
Kuala Lumpur, Malaysia	124	56 (45.2)	G1P[8] (n = 40)G1P[nt] (n = 14)G2P[nt] (n = 1)G3P[nt] (n = 1)	8 (6.5)	GII.na (n = 1)GII.2 (n = 5)GII.4 (n = 1)“GII.13 (n = 1)
Tunis, Tunisia	92	19 (20.7)	G3P[8] (n = 19)	10 (10.9)	GI.3 (n = 3)GII.na (n = 1)GII.2 (n = 4)GII.3 (n = 2)
Cayenne, French Guiana	84	2 (2.4)	G2P[4] (n = 1)G2P[nt] (n = 1)	11 (13.1)	GI.3 (n = 4)GII.4 (n = 4)GII.5 (n = 2)GII.17 (n = 1)
Hong Kong, China	75	32 (42.7)	G1P[8] (n = 6)G3P[8] (n = 23)G2P[nt] (n = 1)G5P[nt] (n = 1)G9P[nt] (n = 1)	9 (12.0)	GI.6 (n = 1)GII.3 (n = 3)GII.4 (n = 1)GII.6 (n = 2)GII.7 (n = 1)GII.14 (n = 1)
Entebbe, Uganda	53^a^	9 (17.0)	G1P[6] (n = 2)G1P[8] (n = 2)G1P[nt] (n = 1)G2P[4] (n = 1)^a^G2P[6] (n = 1)G3P[8] (n = 2)	4 (7.5)	GI.na (n = 1)GI.5 (n = 1)^a^GII.6 (n = 1)GIV.1 (n = 1)
Bangui, Central AfricanRepublic	17	5 (29.4)	G1P[6] (n = 3)G1P[8] (n = 1)G3P[8] (n = 1)	3 (17.6)	GI.3 (n = 1)GII.2 (n = 1)GII.3 (n = 1)
Dakar, Senegal	10	3 (30.0)	G1P[6] (n = 3)	2 (20.0)	GII.6 (n = 2)
Kalubowila, Sri Lanka	9	4 (44.4)	G1P[8] (n = 2)G2P[4] (n = 1)G4P[nt] (n = 1)	0	not detected
Outram, Singapore	9	1 (11.1)	G1P[8] (n = 1)	0	not detected
Seoul, South Korea	7	6 (85.7)	G1P[8] (n = 5)G2P[4] (n = 1)	0	not detected
Kinshasa, DemocraticRepublic of the Congo	5	3 (60.0)	G1P[8] (n = 3)	0	not detected
Total	485	140 (28.9)	G1P[6] (n = 8)G1P[8] (n = 60)G1P[nt] (n = 15)G2P[4] (n = 4)G2P[6] (n = 1)G2P[nt] (n = 3)G3P[8] (n = 45)G3P[nt] (n = 1)G4P[nt] (n = 1)G5P[nt] (n = 1)G9P[nt] (n = 1)	47 (9.7)	GI.3 (n = 8)GI.5 (n = 1)GI.6 (n = 1)GI.na (n = 1)GII.na (n = 2)GII.2 (n = 10)GII.3 (n = 6)GII.4 (n = 6)GII.5 (n = 2)GII.6 (n = 5)GII.7 (n = 1)GII.13 (n = 1)GII.14 (n = 1)GII.17 (n = 1)GIV.1 (n = 1)

NOTE.

a Two of the 53 fecal specimens collected from Entebbe, Uganda were obtained from adults involved with specimen collection in the area: specimens E56 (rotavirus G2P[4]) and E57 (norovirus GI.5).

b Genotypes for 18 noroviruses were polymerase-based alone, because they could not be determined by the capsid-based genotyping method: four GII.4 noroviruses from French Guiana; one GII.6 norovirus from Uganda; one GII.4 norovirus and one GII.6 norovirus from China; two GII.4 noroviruses and one GII.13 norovirus from Malaysia; two GI.3 noroviruses, four GII.2 noroviruses, one GII.3 norovirus, and one GII.4 norovirus from Tunisia.

The retrospective research use of the samples in this study was approved by the NIAID Institutional Review Board (protocol 10-I-N094); a waiver of informed consent was granted for this use.

### Viral RNA Extraction and Genotyping

Viral RNA was extracted from stool specimens with the MagMAX™ Viral RNA Isolation Kit (Life Technologies Corp., Carlsbad, CA) in a MagMAX™ Express Magnetic Particle Processor (Applied Biosystems, Carlsbad, CA).

Rotavirus screening was initiated by a previously described quantitative RT-PCR method that targets a conserved portion of gene NSP3 (rotavirus nonstructural protein 3) [23]. Rotavirus-positive specimens were selected for VP7 and VP4 genotyping using previously described methods [26]–[29] (Text S1). All products of VP7 and VP4 genotyping reactions were resolved on 3.0% SeaKem® LE agarose (Lonza Group Ltd., Basel, Switzerland) gels in 1X tris-acetate buffer containing 1 µg/mL ethidium bromide. When the VP4 genotyping reaction was negative for a rotavirus-positive specimen, the VP4 genotype was designated as “not typed” (“nt”).

Standard RT-PCR followed by sequencing was used to detect and genotype noroviruses. Diagnostic primers that anneal to highly conserved portions of the RdRp were used in the form of primer pools in a one-step RT-PCR amplification of RNA (SuperScript® III One-Step RT-PCR System, Life Technologies Corp.) [25]. After the amplified RdRp region was sequenced, the genotype was confirmed by performing two additional RT-PCR reactions with primer pools that targeted conserved regions of GI or GII norovirus capsid genes [24]. Products of amplification were resolved on 1.5% SeaKem® LE agarose (Lonza Group Ltd.) gels as described above, and bands were excised from the gel and purified with the QIAquick® Gel Extraction Kit (Qiagen, Valencia, CA).

### Sequence Analysis of PCR Product

Sequencing was performed on gel-purified DNA amplicons with reagents in the BigDye® Terminator V3.1 Cycle Sequencing reaction kit (Life Technologies Corp.) and with the diagnostic RT-PCR primers. The reactions were analyzed in an automated 3730 DNA Analyzer (Life Technologies Corp.). Resulting sequences were entered into a BLASTn search to identify homologous sequences [5], [13]. Each norovirus was assigned a genotype based on a partial capsid sequence, unless only a partial polymerase sequence was obtainable (Table 1). Norovirus genotypes that could not be grouped with an existing defined genotype were designated as “not assigned” (“na”) [13].

To obtain sequences for phylogenetic analysis, a SuperScript® III One-Step RT-PCR System with Platinum® *Taq* High Fidelity (Life Technologies Corp.) was used to amplify full-length norovirus VP1 and rotavirus VP7 capsid genes from strains with RNA of sufficient quality. Additional VP1 and VP2 genomic sequences were determined for two norovirus strains of particular interest: Hu/NoV/KL45/Malaysia/1978/GII.na and Hu/NoV/T091/Tunisia/1976/GII.na. Diagnostic primers, as well as newly designed primers, were used in various combinations to determine a consensus sequence for the entire gene, excepting the GIV.1 norovirus, for which only a partial capsid sequence (360 bp) was obtained (Table S1). GenBank accession numbers for all capsid gene sequences obtained in this study are as follows: JN699033–JN699050 and JN989560 (Table S2).

### Phylogenetic Analysis of Rotavirus and Norovirus Sequences

Nucleotide sequences were aligned using Clustal X 2.1, and alignments were manually edited in MegAlign version 9.0 (Lasergene, Madison, WI) [30]. For maximum likelihood phylogenetic analyses, parameter values for best-fit evolutionary models of nucleotide substitutions were determined using Akaike information criterion (AIC) as implemented in MODELTEST [31]. Phylogenetic trees were inferred by maximum likelihood reconstruction of sequence alignments using PhyML software in the context of evolutionary models [31], [32]. Trees were reconstructed with 500 bootstrap pseudoreplicates for statistical reliability. All trees were visualized and annotated using Fig Tree software (http://tree.bio.ed.ac.uk/software/figtree/).

Nucleotide (nt) and amino acid (aa) variations between selected sequences (percent nt, aa distance) were performed using the Tamura-Nei model and Poisson correction, respectively (MEGA version 5), using multiple sequence alignments generated with Clustal X 2.1 [30], [33]. When determining the non-identical residue variations between a single sequence as compared with the remainder of its phylogenetic cluster, individual sequences or groups of sequences were defined as “taxa” and an average distance between groups was calculated.

Bayesian evolutionary analyses were performed separately for VP1 gene sequences of GI.1 and GI.3 noroviruses, but not for rotaviruses, as a Bayesian analysis for G9 and G12 rotaviruses has been published recently [8]. Our GI.3 subset in the analysis contained three sequences obtained from this study (Hu/NoV/B8/CentralAfricanRepublic/1977/GI.3, Hu/NoV/C9/FrenchGuiana/1978/GI.3, and Hu/NoV/C91/FrenchGuiana/1978/GI.3) in addition to sequences available in GenBank, whereas our GI.1 subset contained only GenBank sequences (Table S3). GI.1 norovirus sequences were used as a control, because they belong to Genogroup GI, and the GI.1 sequences available in GenBank had a wide-range of collection dates (1968–2007), similar to those of GI.3 (1977–2007). Sequence alignments and best-fit models of nucleotide substitutions were obtained as described above. Specimen collection dates and prior values associated with best-fit models of nucleotide substitutions were entered into the BEAUTi component of the BEAST software package [34]. Bayesian skyline models for population growth and rates of nucleotide substitution were estimated using Bayesian Markov chain Monte Carlo (MCMC) as implemented in BEAST [34], [35]. Strict, relaxed uncorrelated lognormal, and relaxed uncorrelated exponential clock models were used, and each BEAST file generated in BEAUTi was independently run three times [36]. Chains were run until convergence was reached, and effective sampling size (ESS) values for all parameters were greater than 200, as determined by TRACER analysis software of the BEAST package [34]. Statistical reliability was verified by the presence of large ESS values for all parameters in our BEAST analyses, and consistent rates of evolution across multiple BEAST runs.

## Results

### Relative Detection of Rotaviruses and Noroviruses

**Figure 1:**
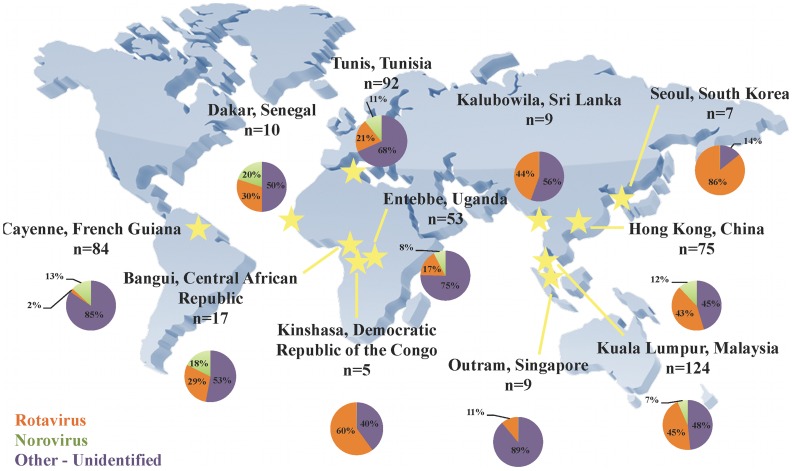
Map of the relative frequencies of rotaviruses and noroviruses detected in each region of this study.

A total of 485 diarrheic stools obtained in a multi-center epidemiologic study in 1976–1979 were examined for rotaviruses and noroviruses using quantitative RT-PCR [23] and diagnostic sequencing [24], [25]. Rotaviruses were detected in 140 (28.9%) and noroviruses in 47 (9.7%) of the stools. Eight (1.6%) stool specimens were positive for both rotavirus and norovirus, but no dual rotavirus or dual norovirus infections were found. Rotavirus-positive stools were found in each geographic region, whereas noroviruses were found in all but four regions: South Korea, Singapore, Sri Lanka, and Democratic Republic of the Congo, with only 5–9 specimens collected for each of these countries (Figure 1). In all regions, rotaviruses outnumbered noroviruses, with the exception of French Guiana.

### Distribution of Viral Genotypes

The genotypes of rotaviruses and noroviruses were determined [26]–[28] and analyzed for their prevalence and global distribution in the 1970’s. All noroviruses were genotyped according to their viral polymerase and/or capsid, and all rotaviruses by their VP7, but only 122/140 (87.1%) could be genotyped for VP4. Noroviruses characteristically showed a diversity of genotypes in each setting. Rotavirus genotypes were either diverse or dominated by a single genotype in each setting. For example, several genotypes (G3P[8], G2P[nt], G5P[nt], and G9P[nt]) were found in 32 rotavirus specimens from Hong Kong, while only one genotype (G3P[8]) was detected in 19 rotavirus specimens from Tunisia (Table 1).

**Figure 2:**
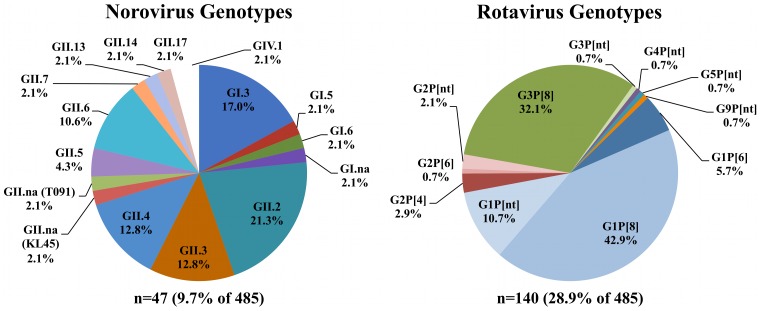
Overall distribution of rotavirus and norovirus genotypes detected in this study.

Rotavirus genotype G1P[8] predominated worldwide (n = 60), and G3P[8] was the second-most prevalent (n = 45) (Figure 2). Rotaviruses detected in this global cohort generally followed characteristically observed G–P genotype combinations, with the exception of a G2P [6] strain detected in Uganda. The G2P [6] combination is characteristically considered to be atypical, but has been detected in Africa at increasing frequencies in recent years [4], [6], [37]. Two interesting G-types were found in Hong Kong: G5, a G-type more commonly seen in farm animals, and hypothesized to have arisen from a natural reassortment event between animal and human rotavirus strains [6], [38]–[40]; and G9, a G-type emerging in prevalence since the mid-1990’s [6]–[8].

The most common circulating norovirus genotypes were GII.2 (n = 10), GI.3 (n = 8), GII.4 (n = 6), although representative noroviruses from a large variety of other genotypes were also detected (Figure 2). A single GIV.1 genotype was detected in a specimen from Uganda from 1976, which predates the oldest known GIV.1 strains from 1998.

### Phylogenetic Relationship of Archival Viruses with Current Strains

**Figure 3:**
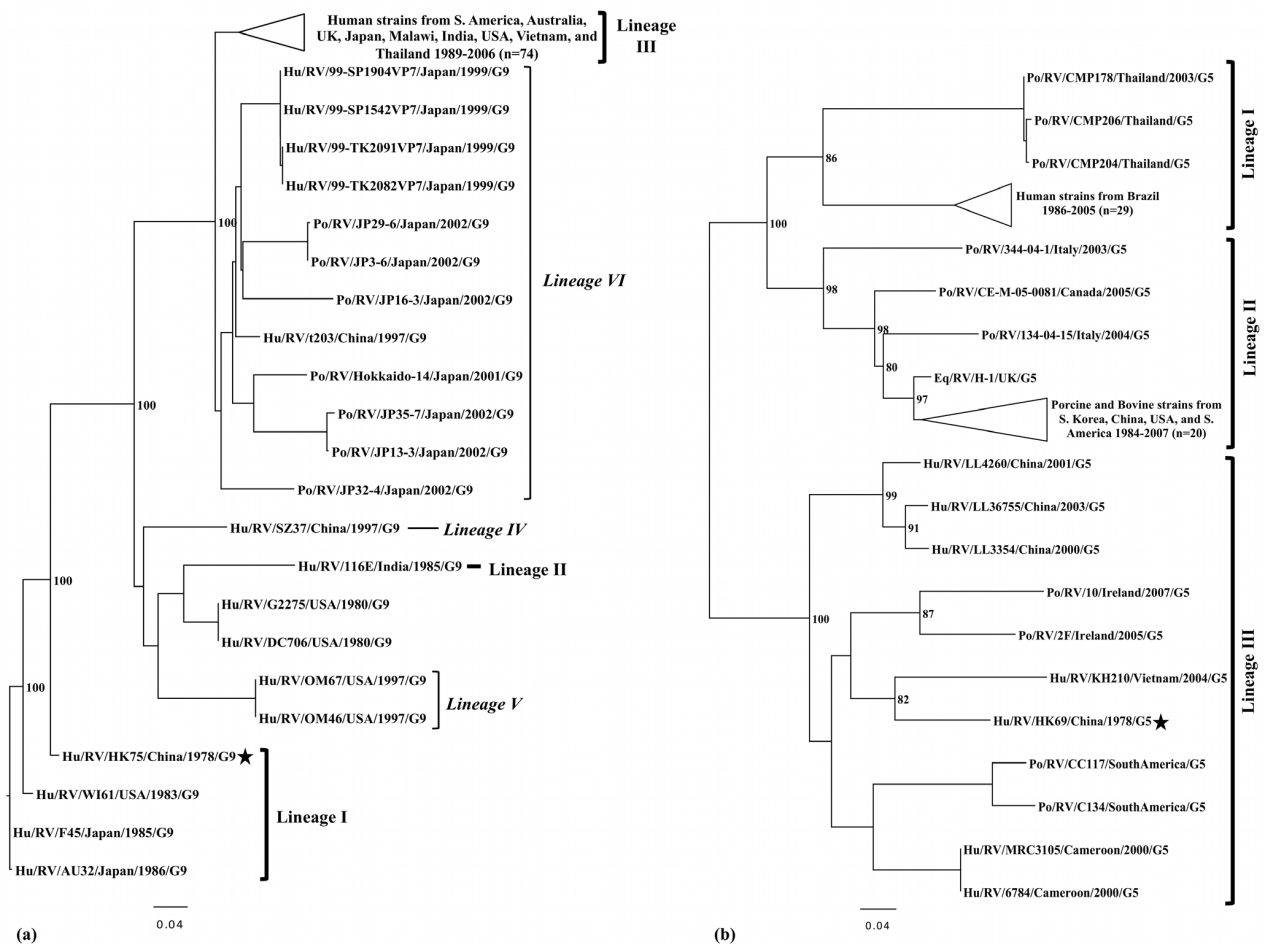
Phylogenetic analyses of partial VP7 capsid nucleotide sequences from rotaviruses. Bootstrap values are shown as percent values for major nodes, and large clusters are collapsed. Black stars indicate sequences obtained from this study. (a) Inferred phylogeny for G9 rotaviruses as determined by a maximum likelihood phylogenetic analysis, including previously defined lineages. Bold brackets indicate consistently defined lineages I, II, and III. Subclusters within lineage III, described by Matthijnssens *et al.*, are indicated in italics, and lineages IV, V, and VI, described by Martinez-Laso *et al.*, are also listed. Strains Hu/RV/G2275/USA/1980/G9 and Hu/RV/DC706/USA/1980/G9 are of indeterminate lineage. (b) Inferred phylogeny for G5 rotaviruses as determined by a maximum likelihood phylogenetic analysis, including previously defined lineages.

**Figure 4:**
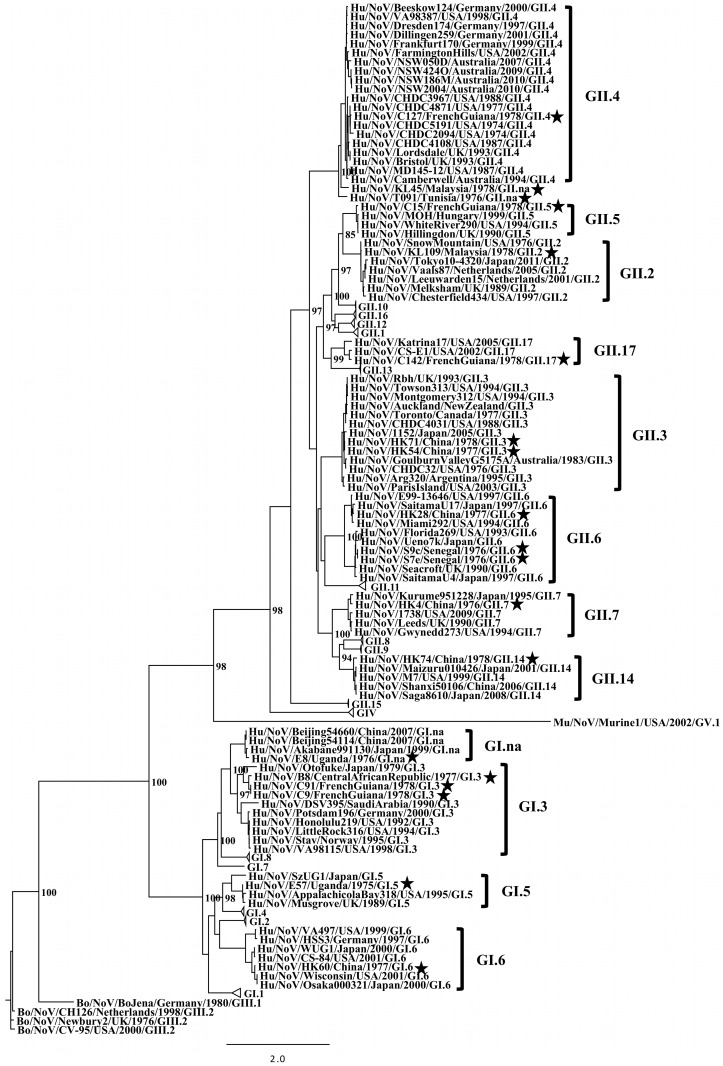
Maximum likelihood phylogenetic analysis of full-length VP1 capsid nucleotide sequences from noroviruses. Bootstrap values are shown as percent values for major nodes. Black stars indicate sequences obtained from this study. Bold brackets indicate genotypes with representatives from this study, and genotypes without representatives from this study are collapsed. Animal sequences representing additional genogroups GIII and GV are also provided.

**Figure 5:**
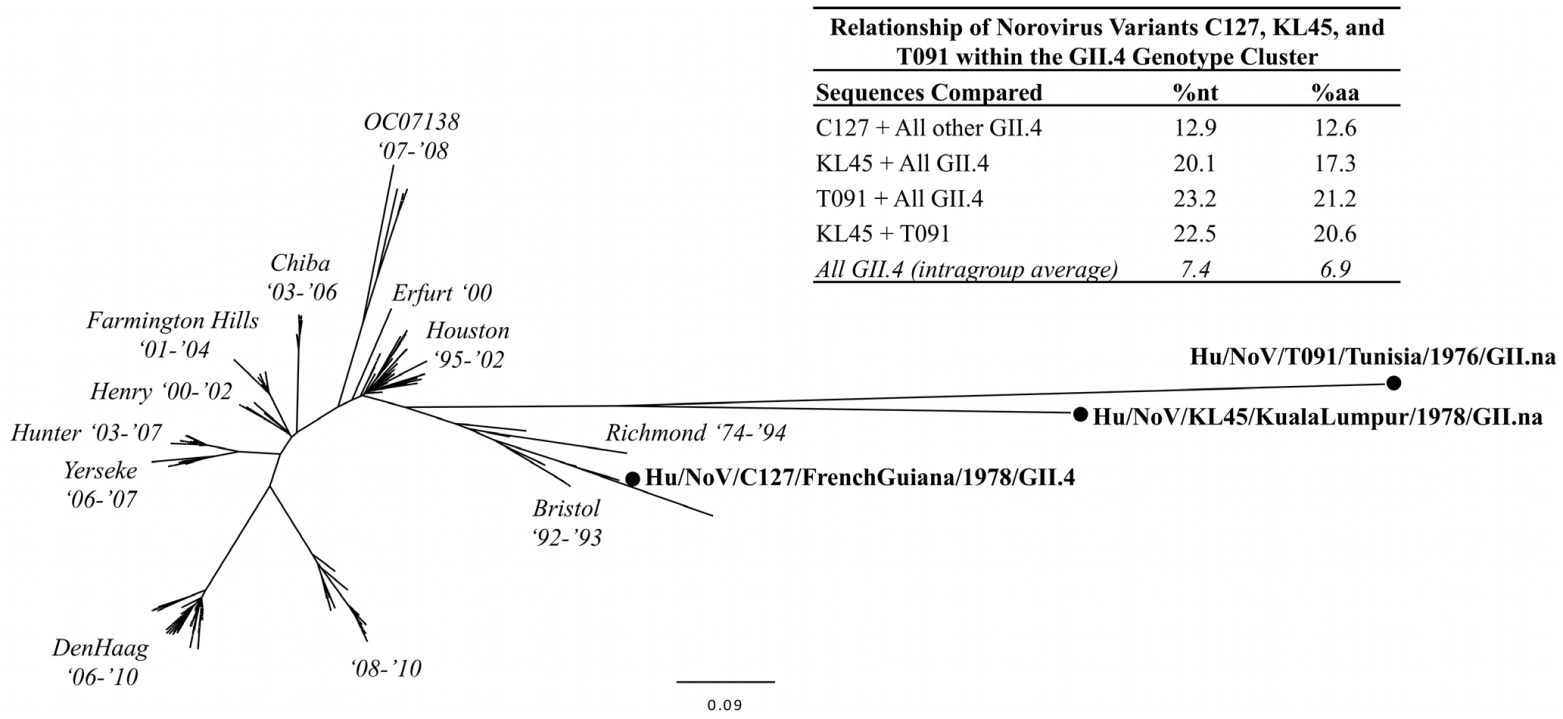
Maximum likelihood phylogenetic analysis of full-length VP1 capsid nucleotide sequences from all GII.4 variant clusters (n = 162 sequences). Variant clusters described by Zheng *et al.* are indicated in italics along with their associated years of specimen collection. A new cluster was defined (“08–10”), which contains strains obtained from the United States, Australia, Europe, Asia, and Africa. Percent nucleotide and amino acid distances are provided in an inlaid table. These distances were determined from a multiple sequence alignment containing all known GII.4 nucleotide sequences available in GenBank as of April 2012 (n = 663 sequences). An average intragroup distance was calculated for all GII.4 sequences, excluding the unique genogroup GII variants KL45 and T091.

**Figure 6:**
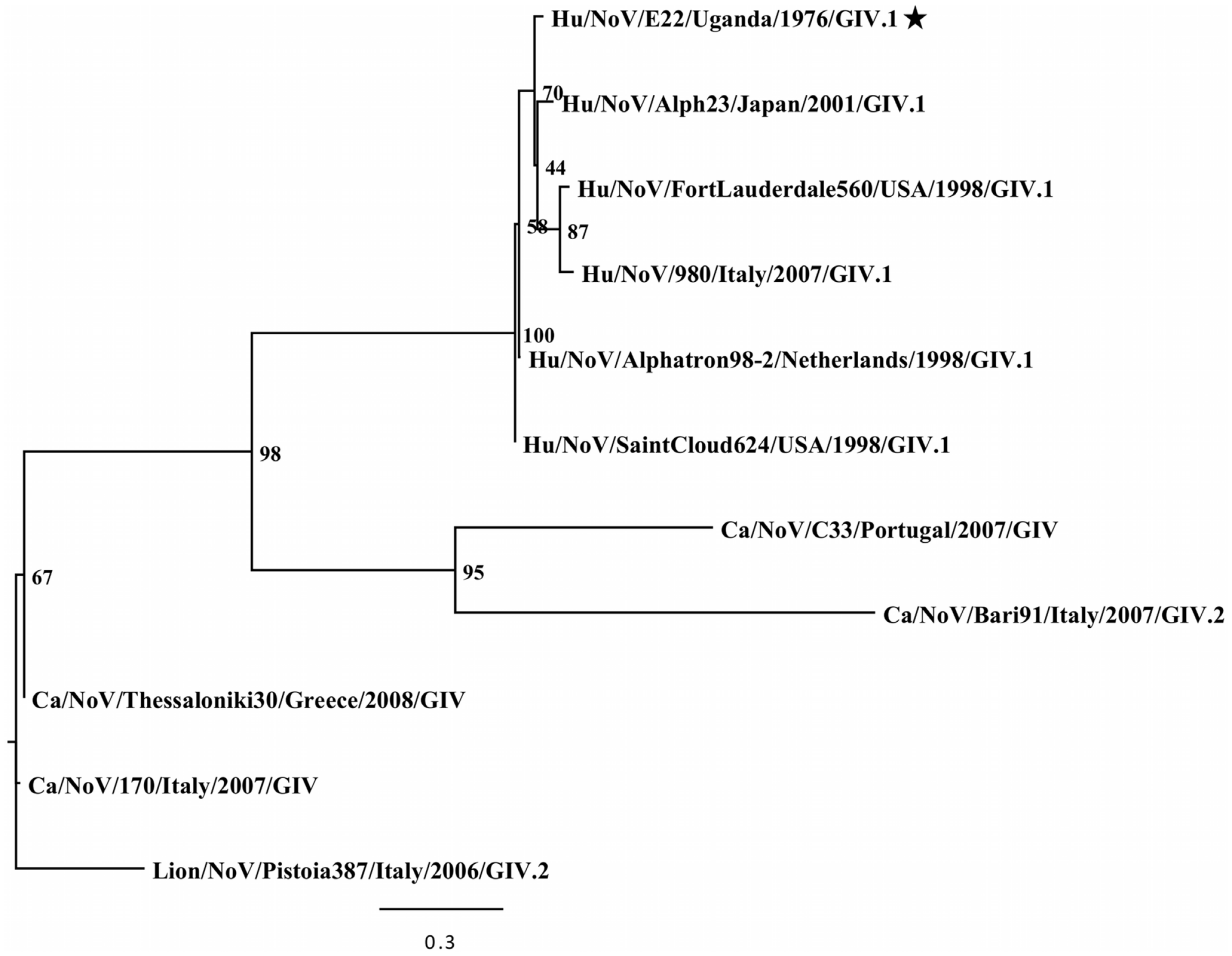
Maximum likelihood phylogenetic analysis of partial VP1 capsid sequences from GIV noroviruses. Bootstrap values are shown as percent values for major nodes. A black star indicates the sequence obtained from this study.

Phylogenetic analyses were performed with the full-length VP7 capsid gene of two rotaviruses detected in this study, the full-length VP1 capsid gene of 19 noroviruses, and the partial VP1 capsid gene of a GIV.1 norovirus (Figures 3, 4, 5, 6).

A G9 rotavirus was detected in a stool collected in Hong Kong in 1978 (Hu/RV/HK75/China/1978/G9), which was of particular interest because G9 rotaviruses emerged in prevalence during the 1990’s [4], [6]–[8]. In an evolutionary analysis, HK75 clustered most closely with Lineage I of the three previously described lineages of G9 rotaviruses (3.1% nt, 2.2% aa distance) [8], [41] (Figure 3a). Additional lineages have also been described [42]. Lineage I includes several of the oldest known G9 rotaviruses, which were detected in fecal specimens from the 1980’s.

A G5 rotavirus was detected in a stool collected in Hong Kong in 1978 (Hu/RV/HK69/China/1978/G5), making it the oldest identified G5 rotavirus thus far. Strain HK69 clustered within Lineage III of three previously defined G5 lineages (13.1% nt, 4.9% aa distance) [39] (Figure 3b). Interestingly, HK69 did not cluster with older human G5 strains that were detected in Brazil in a closer time frame (late 1980’s–1990’s), but instead with an Asian strain from 2004, Hu/RV/KH210/Vietnam/2004/G5 (12.0% nt, 6.3% aa distance).

Several norovirus genotypes were detected in this study, including at least one representative of each genogroup that infects humans (I, II, and IV) (Table 1, Figures 4 and 6). Some of these viruses are the oldest known samples of their respective genogroups and/or genotypes: Hu/NoV/C15/French Guiana/1978/GII.5, Hu/NoV/C142/FrenchGuiana/1978/GII.17, Hu/NoV/HK28/China/1977/GII.6, Hu/NoV/S7e/Senegal/1976/GII.6, Hu/NoV/S9c/Senegal/1976/GII.6, Hu/NoV/HK4/China/1976/GII.7, Hu/NoV/HK74/China/1978/GII.14, Hu/NoV/E8/Uganda/1976/GI.na, Hu/NoV/B8/CentralAfricanRepublic/1977/GI.3, Hu/NoV/C9/FrenchGuiana/1978/GI.3, Hu/NoV/C91/FrenchGuiana/1978/GI.3, Hu/NoV/E57/Uganda/1975/GI.5, Hu/NoV/HK60/China/1977/GI.6, and Hu/NoV/E22/Uganda/1976/GIV.1 (Table 1, Figure 4).

GII.4 noroviruses are the most common genotype associated with epidemic norovirus gastroenteritis [12], [14]–[16]. Two genogroup GII variants that are most homologous with GII.4 noroviruses were detected in diarrheic specimens collected from two infants hospitalized with acute gastroenteritis: Hu/NoV/KL45/Malaysia/1978/GII.na and Hu/NoV/T091/Tunisia/1976/GII.na. GII variants KL45 and T091 could not be clearly assigned to a defined genotype (>14.3% amino acid distance in VP1) and were designated “GII.na”(Figures 4 and 5) [13]. Strain KL45 was detected in a stool specimen obtained from a 9 month-old Malaysian male infant on the fourth day after illness onset, and strain T091 was detected in a stool specimen obtained from a 3 month-old Tunisian female infant on the sixth day after illness onset. The Malaysian infant resided in a rural setting and had 10 episodes of diarrhea per day while hospitalized, whereas the Tunisian infant resided in an urban setting and had 5 episodes of diarrhea per day while hospitalized.

In addition, the full-length VP1 capsid sequence was obtained for a representative GII.4 norovirus (12.9% nt, 12.6% aa distance) detected in a stool specimen from Cayenne, French Guiana within the same time frame: Hu/NoV/C127/FrenchGuiana/1978/GII.4 (Figures 4 and 5). In an evolutionary analysis of all GII.4 variants described by Zheng *et al.* [43], in addition to a new cluster of strains from 2008–2010, strain C127 clustered with the oldest GII.4 noroviruses from 1974–1994, whereas strains KL45 and T091 were the most distant species when compared to the rest of the GII.4 cluster (KL45: 20.1% nt, 17.3% aa distance; T091: 23.2% nt, 21.2% aa distance) (Figure 5). Furthermore, strains KL45 and T091 were genetically distinct from each other (22.5% nt, 20.6% aa distance).

A single GIV.1 norovirus (Hu/NoV/E22/Uganda/1976/GIV.1) was detected in a stool collected from a child in Entebbe, Uganda in 1976, which predates the oldest known GIV.1 strains from 1998. In an evolutionary analysis of a partial capsid sequence (360 bp), E22 clustered with contemporary human GIV.1 strains (7.3% nt, 3.0% aa distance) (Figure 6).

### Norovirus GI.3 and GI.1 Nucleotide Substitution Rates

Rates of nucleotide substitution/site/year were estimated for GI.1 and GI.3 noroviruses by analysis of VP1 gene sequences (Table S3). We compared the rates of nucleotide substitution/site/year using strict, relaxed uncorrelated lognormal, and relaxed uncorrelated exponential clock models. For each clock model, we found consistent rates of 1.25–3.52×10^−3^ (95% HPD range of 0.0559–6.11×10^−3^, collectively) nucleotide substitutions/site/year (Table 2).

**Table 2:** Nucleotide Substitution Rates for the VP1 Gene of GI.3 and GI.1 Noroviruses^a^.

Norovirus Genotype	Molecular Clock	Nucleotide substitution rate in 10^−3^ substitutions/site/yr and (95% HPD)^b^
GI.3	Strict	1.25 (0.240–2.25)
	Uncorrelated lognormal	1.70 (0.0559–3.48)
	Uncorrelated exponential	2.56 (0.0782–6.11)
GI.1	Strict	1.37 (0.676–2.03)
	Uncorrelated lognormal	1.74 (0.538–3.95)
	Uncorrelated exponential	3.52 (0.713–6.02)

Note.

a The nucleotide substitution rate is the mean rate for three individual BEAST runs.

b Values in parentheses are the upper and lower bounds of the 95% highest posterior density interval.

## Discussion

Rotaviruses and noroviruses are important causative agents of childhood diarrhea, particularly in developing countries [2], [3]. Following the discovery of rotaviruses in 1973 [18] and noroviruses in 1972 [17], the LID collaborated with the WHO to assess the role of viruses in childhood diarrhea [19]. With consistent evidence that rotaviruses were a leading cause of diarrhea worldwide, the WHO recommended rotavirus vaccine initiatives as a global health priority [19], [44]. Norwalk virus was not detected in infants and young children in these early studies, although our current study supports an emerging consensus that noroviruses are second in importance to rotaviruses as agents of severe pediatric gastroenteritis [3].

Our analyses of these archival fecal specimens revealed that both rotaviruses and noroviruses circulated throughout the developing world in the late 1970’s. Rotaviruses were generally more prevalent than noroviruses, with the exception of Cayenne, French Guiana. The most common circulating rotavirus and norovirus genotypes were consistent with those generally observed to be most prevalent today (i.e. GII noroviruses and G1 rotaviruses) [4], [6], [12], [14]–[16], and co-infections with representatives of both viruses were observed in multiple regions.

Certain rotavirus strains detected in these archival specimens are of particular interest as they represent unusual VP7 genotypes or VP7/VP4 genotype combinations. One is a G2P[6] rotavirus detected in a child in Entebbe, Uganda in 1976. The first G2P[6] genotypic combination (strain 1076) was isolated from a neonate in Sweden with an asymptomatic rotavirus infection in 1975 [45]. Our identification of this combination in Entebbe indicates that both asymptomatic and symptomatic rotavirus infections were associated with the G2P[6] genotypic combination that circulated in different parts of the world in that time period. Moreover, in a review of studies published during 1997–2006, the G2P[6] genotype was detected in 10% of African children with acute gastroenteritis, indicating that this strain has become established in more recent years in Africa [37].

In addition, G5 and G9 rotavirus strains were detected in a 4.5 month old and a 5 month old infant, respectively, in Hong Kong, China in 1978. G9 rotaviruses have been emerging in prevalence globally since the mid-1990’s [6]–[8], but the detection of Hu/RV/HK75/China/1978 indicates that this genotype has been circulating since at least 1978 in Asia. The VP7 gene of strain HK75 clustered most closely with those of the oldest lineage (I) of G9 rotaviruses, which no longer are known to circulate today, as they were replaced over time by phylogenetic lineages comprised of contemporary strains (II and III) [8], [41], [42]. HK75 is also the oldest known Lineage I strain and the immediate precursor to all contemporary lineages. It is of interest that Lineage I G9 strains have been shown to exhibit the broadest neutralizing cross-reactivity, as they neutralize Lineage II and III G9 strains to high titer, making them ideal vaccine candidates [46]. Previous analyses of G5 rotaviruses suggested that zoonotic or natural reassortment events may have given rise to G5 diversity [38]–[40]. Our analysis confirmed the presence of three previously defined G5 lineages [39], each associated with at least one zoonotic event. All human strains clustered within two lineages (I and III), and both lineages also contained porcine strains, but no other types of animal strains, suggesting that swine may be involved with zoonotic transmission to humans. Lineage II contained no human strains, but closely related animal strains (swine, cattle, and horses). Strain HK69 predates the oldest known human G5 strains from Brazil [38], which indicates that any natural reassortment or zoonotic event may have occurred as early as 1978.

This study identified a variety of norovirus genotypes, and an evolutionary analysis of full-length norovirus VP1 genes illustrated this diversity. Norovirus genotypes from this cohort were diverse globally, indicating no evident trend in distribution. A previous evolutionary analysis of GII.3 noroviruses suggested that, although these viruses accumulate mutations over time, they tend to eventually revert back to the amino acid composition of older strains [47]. Our analysis supports this finding and suggests that other norovirus genotypes may exhibit similar characteristics (GI.3, GI.5, GI.6, GII.6, GII.7, and GII.17).

Genotype GII.4 noroviruses are the most common strains associated with outbreaks of norovirus gastroenteritis worldwide [12], [14]–[16], [43]. However, GII.4 noroviruses detected in this study (n = 6) were outnumbered by GII.2 (n = 10) and GI.3 (n = 8) noroviruses, an observation that could reflect sampling procedures or the distribution of genotypes at that time. The original objective of this study was to document cases of viral associated diarrhea in specific settings globally, and not necessarily to characterize outbreaks. One of the six GII.4 norovirus strains detected in this collection (Hu/NoV/C127/FrenchGuiana/1978/GII.4) had sufficient RNA quality for full-length VP1 capsid sequencing and phylogenetic analysis; this strain clustered with the oldest GII.4 variants from Children’s Hospital, Washington DC (1974–1977) [48]. However, two additional genogroup GII variants with closest homology to the GII.4 genotype (Hu/NoV/KL45/Malaysia/1978/GII.na and Hu/NoV/T091/Tunisia/1976/GII.na) were also detected in specimens collected in a similar time frame, but did not meet the criterion for belonging to GII.4 (>14.3% aa distance) [13]. Furthermore, strains KL45 and T091 were of sufficient sequence dissimilarity for each to be proposed as a new genotype (>14.3% aa distance) [13]. These GII variants have no contemporary counterparts, since none were available in GenBank, despite the ability to detect any closely homologous variants using the standard, widely used norovirus diagnostic primers employed in this study. Thus, strains KL45 and T091 came from unknown sources and caused single episodes of norovirus gastroenteritis without being part of the diverse repertoire of epidemiologically relevant noroviruses that still circulate today. Each of these viruses was detected in stools collected within one week of illness onset, making it unlikely that they arose from multiple mutations obtained during a prolonged infection. Since GII.4 noroviruses, the most homologous species to KL45 and T091, cause outbreaks associated with novel variants that displace others from previous years [43], it is possible that KL45 and T091 represent genetic variants that never became established.

A GIV.1 norovirus was detected in a stool obtained from a child in Entebbe, Uganda in 1976 (Hu/NoV/E22/Uganda/1976/GIV.1), making it the oldest known GIV.1 virus. In an evolutionary analysis, E22 clustered with other human GIV.1 noroviruses. GIV noroviruses are commonly associated with infections in animals, including dogs and lions, and are suggested to have arisen in humans from interspecies transmission due to the genetic similarity between human and animal strains [49]. The 1976 date of collection for strain E22 indicates that GIV noroviruses have been circulating in humans on multiple continents for over 30 years.

Although this study contained a majority of GII noroviruses, we found a large number of GI.3 noroviruses as well. Rates of nucleotide evolution have not been reported previously for GI noroviruses, which circulate at lower frequencies [15], [16], [47], [48]. The nucleotide substitution rates for GI.3 and GI.1 noroviruses are similar for each clock model, which are consistent with traditional rates of evolution for RNA viruses [50], and with previously described rates for GII.3 and GII.4 noroviruses [47], [48]. The comparability in nucleotide evolutionary rates for GI and GII noroviruses indicates that nucleotide evolution is likely not responsible for the higher circulating frequencies of GII versus GI noroviruses.

Rotaviruses have maintained a dominance of the G1 genotype, and other minor genotypes have persisted in humans at lesser frequencies. Conservation of this limited pool of VP7 and VP4 genotypes suggests that multivalent vaccines may be necessary to obtain optimal protection against rotavirus gastroenteritis, and once formulated may persist in their effectiveness. For example, the oldest G9 rotaviruses belonging to Lineage I neutralize more contemporary strains of Lineages II and III, and therefore certain older rotavirus strains may be ideal candidates for attenuation and incorporation into vaccines [46]. Further evidence for the vaccine potential of archival rotavirus strains includes the presence of contemporary G5 rotaviruses that are most closely homologous with older strains, including strain HK69 from this study. The overlapping representation of antigenic VP4 capsid genotypes in rotaviruses with distinct VP7 capsid genotypes, which was demonstrated in this archival study and is also currently observed, further supports rotavirus vaccine development strategies [6], [9], [51]. It is important to note that both multivalent (RotaTeq: attenuated reassortant with human G1, G2, G3, G4, and P[8] antigenicity in a bovine backbone) and monovalent (Rotarix: attenuated human G1P[8]) vaccines have reduced the burden of rotavirus disease in countries where vaccination programs have been implemented [52]. Although the composition of RotaTeq and Rotarix differ both in valency and source of viral strain (i.e. a reassortant with bovine backbone or an attenuated human strain), they have similar efficacies against severe rotavirus gastroenteritis, indicating that the relative importance of VP7 and VP4 antigenicity is unclear [53]. Moreover, the first live, attenuated rotavirus vaccine, RRV-TV, composed of a closely homologous simian backbone strain, including a simian VP4, was efficacious against severe rotavirus gastroenteritis [54]. Regardless of the specificity of vaccine formulations implemented to protect against the conserved VP7 and VP4 genotypes, additional surveillance data will need to be analyzed in the post-vaccine era to determine whether rotavirus evolution differs with the widespread introduction of vaccines [51].

On the other hand, noroviruses exhibit a large reservoir of genetic diversity, and our current understanding of this diversity may be limited, as evidenced by the presence of unique variants (and possibly new genotypes) detected in this cohort (KL45 and T091). Although KL45 and T091 have not been detected in current epidemiological surveys, strain C127 is an archival representative of the highly prevalent contemporary genotype GII.4, indicating that GII.4 noroviruses have maintained importance in the norovirus repertoire over more than 30 years. Thus, vaccination efforts against GII.4 noroviruses may be necessary. Several groups are currently investigating norovirus vaccine formulations for noroviruses and recent advances have been made particularly in GII.4-specific antibody epitope mapping [55]–[60]. However, formulation of a cross-genotype, broad-spectrum norovirus vaccine is complicated by the lack of biological data, namely, it is unknown whether the designated VP1 capsid genotypes correlate with serotype specificity. Some insight was provided by a cross-challenge study in human volunteers, which demonstrated the importance of norovirus genogroups in protecting against disease, when administration of a GI.1 norovirus failed to confer protection upon cross-challenge with a GII.2 norovirus [61]. This suggests that a norovirus vaccine may need to be multivalent, composed of at least three genogroups, which are most divergent among the noroviruses: GI, GII, and GIV.

The preservation of archival specimens by the LID over many years has allowed this retrospective characterization of norovirus and rotavirus molecular epidemiology and study of the evolution from specimens collected in various venues of the developing world in the late 1970’s. This investigation documents that the relative importance of noroviruses as agents of childhood gastroenteritis extends back more than 30 years and also reflects an early underestimation of norovirus incidence when the virus was discovered in the 1970’s. The characterization of rotavirus genotypes and viral lineages provides relevant archival information that supports current efforts towards implementing multivalent rotavirus vaccines.

## Supporting Information

Table S1 Primers Used for Obtaining Full-Length Viral Capsid Gene Sequences for Phylogenetics.
(DOCX)

Table S2 GenBank Accession Numbers and Corresponding Viral Strains.
(DOCX)

Table S3 Sequences of Norovirus VP1 Genes for Bayesian Phylogenetic Analysis of GI.1 and GI.3 Noroviruses.
(DOCX)

Text S1 **Detailed Description of the Rotavirus VP7 and VP4 Genotyping Reactions Used in this Study.**
(DOCX)
