# Supplementary material for: Epidemiology and Evolution of Rotaviruses and Noroviruses from an Archival WHO Global Study in Children (1976–79) with Implications for Vaccine Design
Source: PLoS One. 2013 Mar 25;8(3):e59394. doi: 10.1371/journal.pone.0059394 (PMC3607611; doi:10.1371/journal.pone.0059394)
Supplement: Table S1 — Primers Used for Obtaining Full-Length Viral Capsid Gene Sequences for Phylogenetics. (DOCX) [file pone.0059394.s001.docx]

Supplementary Table 1. Primers Used for Obtaining Full-Length Viral Capsid Gene Sequences for Phylogenetics

| Primer Name | Viral Gene | Primer Sequence (5’-3’) | Citation |
| --- | --- | --- | --- |
| Beg9 | RV VP7 | GGCTTTAAAAGAGAGAATTTCCGTCTGG | Gouvea, V., et al. 1990 |
| End9 | RV VP7 | GGTCACATCATACAATTCTAATCTAAG | Gouvea, V., et al. 1990 |
| TX30SXN | NV VP1 | GACTAGTTCTAGATCGCGAGCGGCCGCCCT_(n=30)_ | Fukushi, S., et al. 2004 |
| 290hijk | NV VP1 | GATTACTCCAGGTGGGACTCCAC (290h); GATTACTCCAGGTGGGACTCAAC (290i); GATTACTCCACCTGGGATTCAAC (290j); GATTACTCCACCTGGGATTCCAC (290k) | Jiang, X., et al. 1999 |
| GIIFnt5100 | NV VP1 | GTGAATGAAGATGGCGTCGAATG | Designed for this study |
| HK46msa2435R | NV VP1 | TGTGTAAAAGGGGTTCACCC | Designed for this study |
| HK46msa2260F | NV VP1 | TACCAGGAATCAGCCCCTGC | Designed for this study |
| HK60msa5283F | NV VP1 | ATGTTCCGCTGGATGCG | Designed for this study |
| E57msa1740R | NV VP1 | TGTTGAAGAGTGAGGTCCTGC | Designed for this study |
| E8msa660F | NV VP1 | GAGGATTGGAAATTTACATCCC | Designed for this study |
| C91msa2330R | NV VP1 | CACAGGCTTTAATTGATAGAAACGG | Designed for this study |
| B8msa5320F | NV VP1 | TCCGCTGGATGCGWTTCC | Designed for this study |
| B8msa6950R | NV VP1 | ACAGGCTTTAATTGATAGAAACGGG | Designed for this study |
| C9msa6820F | NV VP1 | GATACACACCGCAATTTGGG | Designed for this study |
| C9msa5260F | NV VP1 | TAATCCAAGAGGTTAAAGAGGGAGG | Designed for this study |
| B8msa6810F | NV VP1 | TGTTGACCCTGATACACATCGC | Designed for this study |
| GIV.1msaF1370 | NV VP1 | AGTGGTGATGATTCTTTCACCG | Designed for this study |
| GIV.1msaR1843 | NV VP1 | CCCGGATGTRAGGGGCC | Designed for this study |
| GII4cons4510F | NV VP1 | CTGGCTCCTCACHCTCTGTGC | Designed for this study |
| GII4cons6705R | NV VP1 | CAGCAAAGAAAGMTCCAGCCA | Designed for this study |
| KL45F6460 | NV VP1 | ACTTCTACCAAGAAGCTGCCCC | Designed for this study |
| T091F6570 | NV VP1 | ATCACAGTCTCTCACACTGGTCCC | Designed for this study |
