# Supplementary material for: Epidemiology and Evolution of Rotaviruses and Noroviruses from an Archival WHO Global Study in Children (1976–79) with Implications for Vaccine Design
Source: PLoS One. 2013 Mar 25;8(3):e59394. doi: 10.1371/journal.pone.0059394 (PMC3607611; doi:10.1371/journal.pone.0059394)
Supplement: Table S2 — GenBank Accession Numbers and Corresponding Viral Strains. (DOCX) [file pone.0059394.s002.docx]

Supplementary Table 2: GenBank Accession Numbers and Corresponding Viral Strains

| GenBank Accession Number | Rotavirus or Norovirus Strain |
| --- | --- |
| JN699033 | Hu/RV/HK75/China/1978/G9 |
| JN699034 | Hu/RV/HK69/China/1978/G5 |
| JN699035 | Hu/NoV/S9c/Senegal/1976/GII.6 |
| JN699036 | Hu/NoV/S7e/Senegal/1976/GII.6 |
| JN699037 | Hu/NoV/KL109/Malaysia/1978/GII.2 |
| JN699038 | Hu/NoV/HK74/China/1978/GII.14 |
| JN699039 | Hu/NoV/HK71/China/1978/GII.3 |
| JN699040 | Hu/NoV/HK54/China/1977/GII.3 |
| JN699041 | Hu/NoV/HK28/China/1977/GII.6 |
| JN699042 | Hu/NoV/HK4/China/1976/GII.7 |
| JN699043 | Hu/NoV/C142/FrenchGuiana/1978/GII.17 |
| JN699044 | Hu/NoV/C15/FrenchGuiana/1978/GII.5 |
| JN699045 | Hu/NoV/HK60/China/1977/GI.6 |
| JN699046 | Hu/NoV/E57/Uganda/1975/GI.5 |
| JN699047 | Hu/NoV/E8/Uganda/1976/GI.na |
| JN699048 | Hu/NoV/C91/FrenchGuiana/1978/GI.3 |
| JN699049 | Hu/NoV/C9/FrenchGuiana/1978/GI.3 |
| JN699050 | Hu/NoV/B8/CentralAfricanRepublic/1977/GI.3 |
| JN989560 | Hu/NoV/E22/Uganda/1976/GIV.1 |
| JX401279 | Hu/NoV/GII.4/C127/GF/1978 |
| JX401280 | Hu/NoV/GII/KL45/MY/1978 |
| JX401281 | Hu/NoV/GII/T091/TN/1976 |
