# Supplementary material for: Epidemiology and Evolution of Rotaviruses and Noroviruses from an Archival WHO Global Study in Children (1976–79) with Implications for Vaccine Design
Source: PLoS One. 2013 Mar 25;8(3):e59394. doi: 10.1371/journal.pone.0059394 (PMC3607611; doi:10.1371/journal.pone.0059394)
Supplement: Table S3 — Sequences of Norovirus VP1 Genes for Bayesian Phylogenetic Analysis of GI.1 and GI.3 Noroviruses. (DOCX) [file pone.0059394.s003.docx]

Supplementary Table 3. Sequences of Norovirus VP1 Genes for Bayesian Phylogenetic Analysis of GI.1 and GI.3 Noroviruses

| Norovirus Genotype | GenBank Accession Number | Collection Site and Date | Citation |
| --- | --- | --- | --- |
| GI.1 | AY502016 | USA 2001 | Zheng, D. P. and Monroe, S. S., 2006 |
|  | FJ384783 | Sweden 2007 | Nenonen, N. P., et al., 2009 |
|  | EU085522-EU085529 | Sweden 2004 | Nenonen, N. P., et al., 2008 |
|  | M87661 | USA 1968 | Jiang, X., et al., 1993 |
|  | L23828 | Japan 1989 | Wang, J., et al., 1994 |
|  | AB031013 | Japan 1989 | Kobayashi, S., et al., 2000 |
|  | EF547392 | Japan 2003 | Shiota, T., et al., 2007 |
| GI.3 | GQ856470 | China 2007 | Jin, M., et al., 2011 |
|  | GQ856473 | China 2007 | Jin, M., et al., 2011 |
|  | AJ844469 | Japan 2003 | Okada, M., et al., 2005 |
|  | U04469 | Saudi Arabia 1990 | Lew, J. F., et al., 1994 |
|  | AF414403 | USA 1992 | Ando, T., et al., 1995 |
|  | FJ711163- FJ711164 | Sweden 2007 | Nordgren, J., et al., 2010 |
|  | AF414405 | USA 1994 | Ando, T., et al., 1997 |
|  | EF547393 | Japan 2001 | Shiota, T., et al., 2007 |
|  | AB187514 | Japan 1979 | Unpublished, direct submission |
|  | AF439267 | Germany 2000 | Unpublished, direct submission |
|  | AF145709 | Norway 1995 | Myrmel, M. and Rimstad, E., 2000 |
|  | AY038598 | USA 1998 | Jiang, X., et al., 2002 |
|  | AJ277612 | UK 1993 | Green, J., et al., 2000 |
|  | JN699048 | French Guiana 1978 | This study |
|  | JN699049 | French Guiana 1978 | This study |
|  | JN699050 | Central African Republic 1977 | This study |
