# Supplementary material for: Epidemiology and Evolution of Rotaviruses and Noroviruses from an Archival WHO Global Study in Children (1976–79) with Implications for Vaccine Design
Source: PLoS One. 2013 Mar 25;8(3):e59394. doi: 10.1371/journal.pone.0059394 (PMC3607611; doi:10.1371/journal.pone.0059394)
Supplement: Text S1 — Detailed Description of the Rotavirus VP7 and VP4 Genotyping Reactions Used in this Study. (DOCX) [file pone.0059394.s004.docx]

Text S1. A detailed description of the rotavirus VP7 and VP4 genotyping reactions used in this study is as follows: VP7-specific forward and reverse primers (Beg9 and End 9; Gouvea, V., *et al*. 1990) or VP4-specific forward and reverse primers (Con3 and Con2; Gentsch, J. R., *et al*. 1992) were used in a one-step RT-PCR amplification of RNA (SuperScript® III One-Step RT-PCR System, Life Technologies Corp.) to produce a full-length VP7 or VP4 amplicon. A nested second amplification genotyping reaction was performed on all RT-PCR products using an AmpliTaq Gold® 360 Master Mix (Life Technologies Corp.) and pools containing serotype-specific primers to determine VP7 or VP4 genotypes. Each primer pool contained one primer that annealed to a broadly conserved portion of the VP7 or VP4 gene and a collection of genotype-specific primers that annealed to variable regions of the VP7 or VP4 gene to produce amplicons of a characteristic size for each genotype. The VP7 primer pool contained primers 9Con1, 9T-1, 9T-2, 9T-3P, 9T-4, 9T-9B, and FT5 (Gouvea, V., *et al*. 1990; Das, B. K., *et al*. 1994) and the VP4 primer pool contained primers Con3, 1T-1, 2T-1, 3T-1, 4T-1, and 5T-1 (Gentsch, J. R., *et al*. 1992).
